# Synthetic Biology Approaches in the Development of Engineered Therapeutic Microbes

**DOI:** 10.3390/ijms21228744

**Published:** 2020-11-19

**Authors:** Minjeong Kang, Donghui Choe, Kangsan Kim, Byung-Kwan Cho, Suhyung Cho

**Affiliations:** 1 Department of Biological Sciences, Korea Advanced Institute of Science and Technology, Daejeon 34141, Korea; minmin@kaist.ac.kr (M.K.); robinald@kaist.ac.kr (D.C.); kskim2474@kaist.ac.kr (K.K.); 2 Innovative Biomaterials Research Center, KI for the BioCentury, Korea Advanced Institute of Science and Technology, Daejeon 34141, Korea; 3 Intelligent Synthetic Biology Center, Daejeon 34141, Korea

**Keywords:** synthetic biology, genetic engineering, therapeutic molecules

## Abstract

Since the intimate relationship between microbes and human health has been uncovered, microbes have been in the spotlight as therapeutic targets for several diseases. Microbes contribute to a wide range of diseases, such as gastrointestinal disorders, diabetes and cancer. However, as host-microbiome interactions have not been fully elucidated, treatments such as probiotic administration and fecal transplantations that are used to modulate the microbial community often cause nonspecific results with serious safety concerns. As an alternative, synthetic biology can be used to rewire microbial networks such that the microbes can function as therapeutic agents. Genetic sensors can be transformed to detect biomarkers associated with disease occurrence and progression. Moreover, microbes can be reprogrammed to produce various therapeutic molecules from the host and bacterial proteins, such as cytokines, enzymes and signaling molecules, in response to a disturbed physiological state of the host. These therapeutic treatment systems are composed of several genetic parts, either identified in bacterial endogenous regulation systems or developed through synthetic design. Such genetic components are connected to form complex genetic logic circuits for sophisticated therapy. In this review, we discussed the synthetic biology strategies that can be used to construct engineered therapeutic microbes for improved microbiome-based treatment.

## 1. Introduction

In the human body, there are at least as many microbial cells as there are host cells [1]. Microbes, often referred to as microbiota, play various important roles in host functions. According to the ‘hygiene hypothesis’, exposure to various microorganisms is important for the development of the immune system in early childhood [2]. Additionally, many recent studies have revealed the relationship between microbiome dysbiosis and human diseases. For example, the abundance of *Lachnospira*, *Veillonella*, *Faecalibacterium* and *Rothia* species has been shown to be decreased in asthmatic patients compared to that in healthy individuals [3]. As the importance of the relationship between the microbiome and human health is becoming more evident, the use of the microbiome as a therapeutic target is being increasingly highlighted [4]. 

Probiotic therapy is the most representative microbiome-based therapy. Probiotics are bacteria that confer beneficial effects on human health [5]. *Escherichia coli* Nissle 1917 (EcN), isolated from the feces of a soldier who remained healthy during the widespread *Shigella* infection in the midst of World War I, has been commercialized for over a century to treat various enteric diseases. A recently developed therapy called fecal microbiota transplantation (FMT), which involves the transfer of feces from a healthy individual to a patient, is expected to restore and fortify the beneficial microbial communities in human guts afflicted by dysbiosis [6]. Although FMT has been reported as an attractive alternative to conventional therapy with clinically successful examples, there remain critical safety concerns [7].

Naturally occurring microbes have been purposefully manipulated through genetic engineering to improve their therapeutic capability [8]. Engineered microbes can have novel functionalities, such as real-time monitoring of disease progression [9]. Furthermore, targeted therapy through in situ drug bio-production and delivery can reduce adverse side effects while maximizing therapeutic efficacies [10]. In order to modulate the microbes, synthetic biology could be used to develop a strategy to engineer cells with the desired therapeutic functions. In this review, we described synthetic biology approaches for the development of engineered commensal microbes.

## 2. Microbiome Therapy

**Figure 1:**
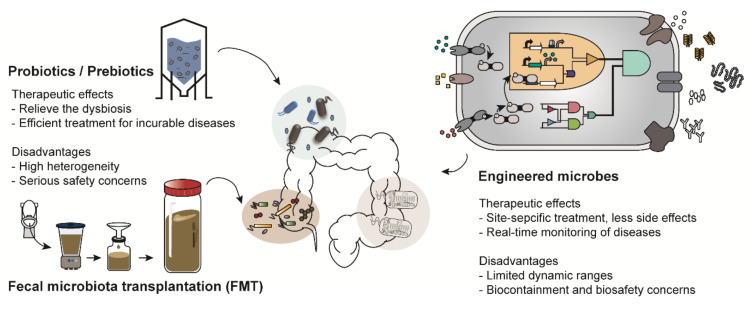
Examples of microbiome-based therapies. Probiotics/prebiotics, administration of microbes that have beneficial effects on human health; Fecal microbiota transplantation (FMT), transfer of the processed feces of healthy people; Engineered microbes, transformation of the genetic platforms to sense and treat the diseases using genetic engineering.

There are various types of microbiome-based therapies, such as probiotic therapy and FMT (Figure 1). Among these, probiotic therapy is one of the most representative microbiome therapies. Probiotics is a collective term for live bacteria that have beneficial effects on human health [5]. Probiotics not only have therapeutic activity but have also been shown to restore a healthy microbial ecology in the body of individuals with dysbiosis [11]. For example, a report showed that constipated patients had significantly decreased numbers of *Bifidobacterium* and *Lactobacillus* compared to those in healthy people and those patients were recovered from constipation by probiotics administration [12]. These strains were also demonstrated to be effective for gastrointestinal diseases such as diarrhea and inflammatory bowel disease [13]. In addition, *Lactobacillus* is an important bacterium commonly found in healthy women; it helps to prevent urogenital infection by maintaining a low vaginal pH [14]. Thus, *Lactobacillus* is frequently used for treatment of vulvovaginal candidiasis [14]. Species of *Bifidobacterium* and *Lactobacillus* are commercially available as nutrient supplements. 

In addition, prebiotic and synbiotic treatments are similar to probiotic therapy. Prebiotics support the growth of beneficial microorganisms in the human body [15]. Synbiotic treatment is a combination therapy that supplies both probiotics and prebiotics simultaneously [16]. However, most probiotics and prebiotics are used as therapeutic supplements rather than medications because of the heterogeneity in their preparation strategy, duration of medication and patients [17]. Most recently, much attention has been paid to FMT owing to its therapeutic efficacy. Donors are selected based on various criteria such as age, body mass index (BMI), history of disease occurrence and drug administration [6]. FMT is mainly employed as an alternative to conventional therapies for patients with incurable diseases such as Parkinson’s disease and chronic intractable constipation [18,19]. However, therapeutic strategies, including probiotic therapy and FMT, have been reported to cause unexpected results and serious safety concerns in a few recently implemented trials [20]. For instance, there was a report in which *Lactobacillus*, generally known to have beneficial effects on enteric diseases, aggravated enteric inflammation, depending on the species used [21]. Furthermore, a patient suffering from acute pancreatitis died after receiving an injection of probiotics due to bowel ischemia [22]. The unexpected results further include systemic infections, deleterious metabolic activities, excessive immune stimulation and gene transfer [23]. Therefore, as part of another microbiome-based therapy, engineered microbes are emerging as a promising alternative [24].

## 3. Synthetic Biology in Diagnosis and Treatment of Human Diseases through Engineered Microbes

**Figure 2:**
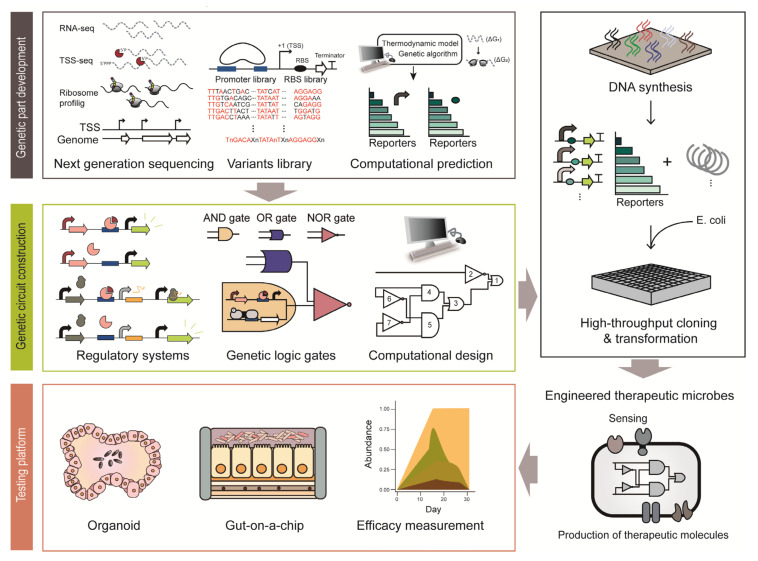
Illustration of the process employed to construct engineered therapeutic microbes based synthetic biology approach.

Synthetic biology provides a strategy to engineer cells with therapeutic functions (Figure 2) [8]. Various genetic platforms to diagnose diseases and deliver therapeutic molecules have been developed and transformed into microorganisms (Figure 2) [24]. Engineered microbes have several advantages compared to conventional therapeutic strategies. For example, engineered microbes can reach specific sites in the human body that conventional drugs have difficulty accessing. In addition, there are fewer side effects because smaller amounts of therapeutic molecules are delivered through engineered microbes in situ, which show similar efficacy as orally administered doses [10]. As some microbes can colonize the human body for a long time, they can be repurposed as monitoring and medicating means for chronic diseases [10]. For example, one of the dominant commensals, *Bacteroides thetaiotaomicron*, persists in the human intestine for more than five years [25]. This section provides an overview of how synthetic biology improves the therapeutic functions of microbes.

### 3.1. Synthetic Biosensors for Detecting Diseases

Various engineered microbes produce and deliver therapeutic molecules to the host for disease treatment (Table 1). However, the production of therapeutic molecules in microbes needs to be carefully controlled because constant production of therapeutics is not only a burden to the endogenous metabolism of microbes but can also cause side effects in the host [10]. One way to solve these problems is to use biosensors that detect specific biomarkers representing or related to disease occurrence and its progression [26]. Biomarkers are available in various forms, such as gases, ions, chemical compounds and biomolecules [27]. For instance, it was reported that patients with enteric inflammation exhibit high concentrations of hydrogen sulfide (H_2_S) and nitric oxide (NO) in their body [28,29]. Also, B-lymphocyte surface antigen B4 (CD19) was designated as a major biomarker for lymphoma, as patients showed high expression levels of B-cell related antigens [30]. 

To develop therapeutic biosensors in microbes, synthetic biology exploits various bacterial sensor systems [79]. There are two bacterial sensing systems, the one-component system (OCS) and the two-component system (TCS) [24]. OCS is a primary bacterial signal transduction system that delivers environmental stimuli to cells [80]. Conversely, TCS consists of two different proteins, a sensor histidine kinase (HK) that senses extracellular stimuli and a response regulator that transduces the signal from the HK to the downstream process [81]. Synthetic biology exploits native bacterial sensor systems to improve the therapeutic efficacy of engineered microbes [10]. One of the simplest biosensors is the nitric oxide (NO) biosensor. NO is a representative biomarker for enteric inflammation [37] and most bacteria harbor various types of NO sensors to detoxify and metabolize NO [82]. Among them, *E. coli* NorR, which is a response regulator that binds to NO and acts as a transcriptional activator of NO reductase, was repurposed as a synthetic NO sensor owing to its high specificity for NO [83]. To visualize the NO sensing, NorR was embedded in the DNA switch composed of FimE-DNA recombinase and fluorescence reporter with reversely oriented promoter at its upstream [37]. Activated by NorR, FimE induces DNA inversion on the reversely oriented promoter of reporter into correct orientation. Thus, presence of NO can be monitored by fluorescence signal [37]. In addition, another biosensor that detects nitrate was shown to serve as an effective indicator of enteric inflammation. The sensor comprises nitrate reductase (Nar) TCS of NarX-NarL and proved its ability to detect nitrate in the dextran sodium sulfate (DSS)-induced mice models [32].

Even when a well-known target biomarker sensor system is available, several engineering processes are required to introduce the desired bacterial sensor into the heterologous host. For example, a TCS-based quorum-sensing (QS) system from *Vibrio cholerae* was transformed into *Lactococcus lactis* to develop a *V. cholerae* infection sensor. In this system, a hybrid histidine receptor was constructed by combining the transmembrane ligand-binding domain of CqsS, which recognizes a cholera autoinducer 1 (CAI-1) and the nisin-controlled expression system from *L. lactis* to detect *V. cholerae*-specific CAI-1 [38]. Similarly, another sensor was developed to detect *Staphylococcus aureus* infection by introducing the accessory gene regulator (Agr) QS system from *Staphylococcus* into *Lactobacillus reuteri*. This system is composed of two genetic modules, a quorum sensor AgrC specific for the *S. aureus* autoinducing peptide I (AIP-1) and a glucuronidase reporter (GusA) under the control of an AgrA-responsive P3 promoter [39]. 

When no sensor systems are available for the target biomarker, synthetic sensors can be designed through computational screening and genetic repurposing. One such example involves the identification of a synthetic biosensor that detects thiosulfate as a biomarker for intestinal inflammation through computational screening [33]. The thiosulfate sensor was found via protein similarity search based on a biosensor that detects a similar molecule, tetrathionate, resulting in 838 candidate sensors from various species provided by UniProtKB [33]. Then, through a series of processes to identify the sensor, the thiosulfate sensing TCS of *Shewanella halifaxensis* was selected and rewired as an enteric inflammatory sensor [33]. As another example, a biosensor for gastrointestinal bleeding was constructed by genetic repurposing of a heme sensor [31]. The heme sensor consisted of the heme-binding transcriptional regulator, HrtR, the HrtR-responsive synthetic promoter P_L(HrtO)_ and the heme transporter, ChuA. As human blood is saturated with heme, it can be readily used as a biomarker to detect internal bleeding. These microbiome-based sensors allow noninvasive and rapid diagnosis of various diseases in situ, compared to conventional endoscopy, sonography or biopsy [33]. 

However, there are several shortcomings in the clinical use of microbial biosensors. First, the dynamic range of sensors should satisfy physiologically relevant levels of biomarkers. Previously, the dynamic range of biosensors has been adjusted by engineering sensor proteins. For example, Abshire et al. increased the sensitivity of heme biosensor by removing partial fragment of the heme-binding domain, which was hindering the energy transfer between the donor and acceptor pair of fluorophore [84]. Second, leaky expression of biosensors may compromise the accuracy of sensor because it can lead to false-positive results [85]. To overcome this problem, a synthetic promoter was developed by replacing the existing operator with a tightly regulated one [86]. Lastly, biosensors should have a high specificity to avoid misdetection of molecules that are structurally analogous to the target molecule. The substrate specificity of biosensor can be engineered by saturation mutagenesis on the ligand binding site of sensor protein. Recent report by Della Corte et al. illustrated this method by engineering the lysine, arginine and histidine-sensing transcriptional regulator LysG to specifically detect only histidine [87].

### 3.2. Synthetic Biology to Deliver Therapeutic Molecules

With a disease biosensor, targeted therapy can be accomplished by spatiotemporally controlled delivery of therapeutic molecules using engineered microbes [24]. There is a wide range of therapeutic molecules for disease treatment, such as therapeutic proteins, biochemicals and antibodies (Table 1). In this section, examples and applications of such therapeutic molecules are described.

#### 3.2.1. Heterologous Production of Host Proteins

Various bioactive molecules are generated by the host, such as signaling molecules, cytokines and enzymes. Most of them are used as effective therapeutic drugs in the clinic [88,89]. Although they show effective therapeutic efficacy at small amounts, technical difficulties remain, such as the lack of effective administration methods and high production costs [41]. In the traditional delivery methods, the therapeutic molecules are easily degraded in the host system such as the acidic stomach [90]. Once small portion of microbes survive through the digestive and immune system of the host, however, it can re-proliferate and deliver the therapeutic molecules directly onto the target site of the human body. In addition, engineered microbes do not go through the endocytosis process due to the oral tolerance in the gut, which is an immune repression against the antigens administered orally [91]. Because the intestine provides the major source of antigens to the gut-associated lymphoid tissue that prevents autoimmune response or inflammation against commensals [92]. Because widely-used engineered microbes are originated from natural human commensals, such as *B. thetaiotaomicron* and *L. lactis*, they can be used for delivery system with minimal immune response by oral tolerance. Therefore, engineered microbes prevents the therapeutic molecules from being exposed to harsh conditions and repress the immune response by administered orally.

Keratinocyte growth factor-2 (KGF-2) is a human growth factor associated with the proliferation of epithelial cells and the intestinal mucosa. It was selected as an effective drug for enteric inflammatory disease; however, low stability in the human body limits its clinical applications [41]. To provide an effective delivery system, an engineered *Bacteroides fragilis* was suggested as a therapeutic chassis [41]. KGF-2 is produced by the xylan inducible promoter from *Bacteroides ovatus* and is secreted via the enterotoxin secretion signal sequence of *B. fragilis* [41]. When xylan was orally administrated in DSS-induced mice models, improvements in colitis were observed, such as repair of damaged epithelium and reduction of rectal inflammation and bleeding [41]. Moreover, another intestine protection molecule, trefoil factor (TFF), was also delivered using live bacteria [93]. TFF stimulates cell migration to repair and protect the damaged intestinal epithelium [94]; however, it is degraded before it can function therapeutically and is removed in the cecum when administered orally [95]. For the active delivery of TFF in situ, an engineered *L. lactis* was used as a delivery vehicle, in which a codon-optimized human *tff* was expressed as a form of Usp45 secretion signal protein-fusion under the lactococcal P1 promoter. TFF secreted in situ activated major therapeutic pathways for the synthesis of prostaglandin-endoperoxide synthase 2, resulting in the healing of colitis in a DSS-induced mouse model [47].

Engineered microorganisms can deliver cytokines that play a central role in modulating the immune response of the human body [96]. Interleukin (IL)-10 is a representative therapeutic molecule delivered through engineered bacteria to treat enteric inflammation [97] and there have been reports using two different methods to produce IL-10 in situ [43,98]. The protective effect of IL-10 produced by engineered bacteria was confirmed in a colitis mouse model and it is in Phase I clinical development [43,44,98]. Moreover, there was a report on generation of another cytokine, transforming growth factor β1 (TGF-β1), through an engineered *E. coli* [40]. The generated cytokine lowered the disease activity index (DAI) score, which includes parameters that determine colitis such as stool frequency, rectal bleeding, endoscopic findings and physician global assessment, in DSS-induced colitis mice [40]. Besides, interleukin-17A (IL-17A) was heterologously produced by *L. lactis* to suppress the incidence of the lung cancer in the TC-1 tumor-bearing mouse model [45]. 

Other microbes producing human lactoferrin (hLF) in response to pathogenic infection have been engineered [48]. hLF acts as an antimicrobial agent by diverse mechanisms such as sequestering free iron and promoting phagocytosis [99]. To supply hLF to the host, the full-length hLF cDNA was expressed through a *Lactobacillus* shuttle vector in *Lactobacillus casei* [48], which showed antibacterial activity by reducing the number of infected *E. coli* in the mouse model.

As illustrated in many examples, engineered microbes enable the delivery of various types of host proteins, such as signal molecules, cytokines and other functional proteins. Engineered microbes can deliver them in active forms while passing through unstable environments of the human body, including the gastrointestinal tract. Therefore, engineered microbes can act as effective platforms to produce and deliver therapeutic host proteins.

#### 3.2.2. Heterologous Production of Therapeutic Proteins

Engineered bacteria that secrete flagellin have been demonstrated to prevent the colonization of enteropathogens [63]. Flagellin is a structural protein of the flagellum, which is an essential component of bacterial motility and facilitates adhesion and invasion of the bacteria in the host. High level of free flagellin in the intestine prevents the adhesion of two enteropathogens, *E. coli* and *Salmonella enterica*, by competitively inhibiting of bacterial attachment to mucin layer. Similarly, flagellin B derived from *Vibrio vulnificus*, has also been shown to repress tumor growth and metastasis. In tumor-bearing mice, the presence of flagellin activates the toll-like receptor 5 (TLR5) signaling pathway to induce an immune response against tumor growth [69]. 

Several engineering attempts have been made to modulate QS in bacteria to prevent pathogen infection. For instance, EcN was engineered to produce pyocin, which acts as DNA-based colicin, in response to the *Pseudomonas aeruginosa* specific autoinducers, acyl homoserine lactones (AHLs) [53,100]. This engineered microbe produces lysis E7 protein and an anti-biofilm enzyme of dispersin B for the effective secretion of pyocin from the cells [53]. Its therapeutic activity which resulted in a reduction of *P. aeruginosa* colonization, was shown in *Caenorhabditis elegans* and mice [53]. Similar to the *P. aeruginosa*-killing engineered microbes, microbes engineered to suppress the virulence of *V. cholerae* in the host have been produced [51]. *V. cholerae* harbors two QS systems, CqsS and LuxPQ receptors. When both the autoinducers are activated simultaneously, the information goes through LuxO to control the level of HapR transcription factor [101]. At low *V. cholerae* cell density, there is no HapR to inhibit the expression of virulence factors, while at high *V. cholerae* cell density accompanied by a high level of CAI-1, virulence gene expression is inhibited [101]. Therefore, by using an engineered EcN that produces high levels of CAI-1, virulence factor production by the pathogen was reduced [51]. Another engineered microbe, which secretes bacterial signal molecule, autoinducer 2 (AI-2) was developed to relieve the streptomycin-induced dysbiosis of gut microbial community [55]. Streptomycin clears most Firmicutes and reduces the Firmicutes/Bacteroides ratio in the afflicted intestine. To resolve this, engineered *E. coli* producing AI-2 was introduced to expand the population of Firmicutes phylum in the host intestine [55].

In addition to signaling molecules, many other functional proteins have been synthesized from engineered microbes for disease treatment. A synthetic hybrid peptide was produced through therapeutic bacteria to inhibit viral infection, such as that of the human immunodeficiency virus (HIV). The engineered bacterium produces an HIV-gp41-hemolysin hybrid peptide, which blocks the fusion of HIV with the CD4 cell membrane for viral invasion of the cell [54]. Furthermore, there are several therapeutic enzymes produced by engineered microbes. Glycosyltransferase mimics the toxin receptor to remove enterotoxins produced by enterotoxigenic *E. coli* and thus, prevent diarrheal disease [57]. The glycosyltransferase delivered through the engineered bacterium showed a significant neutralizing function in the infected rabbit ligated ileal loop model [57]. In addition, superoxide dismutase, which is used to remove the main factors causing reactive oxygen metabolites [50] and linoleic acid isomerase, which alters the composition of host fatty acids to induce anti-obesity effects, have been codon-optimized and delivered into the host using engineered microbes [66]. Also, there was an effort to engineer *E. coli* to produce enzyme myrosinase, which converts host-ingested glucosinolates to sulphoraphane. The resulting metabolite sulphoraphane was shown to exert anticancer effects in murine, human and colorectal adenocarcinoma cell lines [59]. 

As mentioned above, microbes can be engineered to produce several heterologous bacterial proteins in the host. They overcome technical difficulties in delivering these unstable molecules to the host and function as effective therapeutic strains. The delivery efficacy in vivo can be measured in two ways. First, the efficacy to deliver the unstable therapeutic molecules by engineered microbes can be determined by measuring the concentration of the therapeutic molecules produced in vivo. For example, the therapeutic microbes produce AI-2 to treat antibiotic induced gut dysbiosis, where the accumulated AI-2 level in the mice disease model was measured by using biosensor that produce bioluminescence when exposed to AI-2 [55]. Second, the therapeutic effects resulting from the use of engineered microbiomes indirectly proves their delivery efficacy. For example, a lung cancer mouse model administered with engineered *L. latis* secreting IL-17 had a lower tumor incidence rate compared to the group with the wild-type strain [45]. This indicates that the therapeutic molecules are effectively delivered to the target site in vivo. 

#### 3.2.3. Synthetic Metabolism

Engineered microbes can provide synthetic metabolic pathways to repair malfunction or dysfunction of the host metabolism [102]. For example, phenylketonuria is caused by the failure of phenylalanine metabolism. Accumulated phenylalanine in the blood results in serious side effects, such as intellectual disability and seizure [102]. To solve this metabolic syndrome, a synthetic microbe harboring the phenylalanine metabolic pathway was constructed, which exploits two different metabolic enzymes, phenylalanine ammonia lyase (PAL) and l-amino acid deaminase (LAAD), metabolizing phenylalanine to *trans*-cinnamate and phenylpyruvate, respectively [70]. This resulted in the prevention of phenylalanine accumulation and the conversion of phenylalanine to other metabolites through the engineered bacteria [70]. This study reached Phase 1/2a of clinical trial. (Clinicaltrials.gov Identifier: NCT03516487) [70]. As seen in this study, engineered microbes can relieve the malignant effects resulting from the malfunction or dysfunction of the host metabolism by providing synthetic metabolic pathways.

#### 3.2.4. Antigen and Antibody Induction

Engineered microbes can serve as an effective vaccine delivery platform. For example, the autoantigen glutamic acid decarboxylase (GAD65) was expressed in *L. lactis* to treat type I diabetes [76]. Prescribing both GAD65 and IL-10 simultaneously to non-obese diabetic (NOD) mice alleviated the symptoms of insulitis and restored functional β-cell mass and normoglycemia [76]. In addition, to prevent celiac disease, DQ8 gliadin epitopes were produced through engineered microbes to inhibit the local and systemic DQ8-restricted T cell response in NOD mice. Furthermore, antibodies can be delivered through bacteria with an antibody-displaying platform to improve the delivery efficacy of the antibody. For example, HIV-1-specific antibodies were delivered using protein G instead of using soluble antibodies [71]. These examples show the great potential of engineered microbes to deliver both antigens and antibodies into the host for effective treatment.

### 3.3. Genetic Circuits for The Production of Therapeutic Molecules 

Genetic circuits are composed of various genetic parts and regulatory modules to fine-tune the function of therapeutic microbiomes [24]. Using genetic circuits, the gene expression of therapeutic microbiomes can be regulated in a spatiotemporal manner [103]. Bacteria use numerous genetic systems to modulate gene expression in response to a wide variety of external stimuli [104]. Harnessing these regulatory mechanisms, synthetic biologists have constructed genetic circuits to produce desired compounds and novel cellular functions for therapeutic outputs in humans [105]. For example, a dominant commensal, *B. thetaiotaomicron* is regarded as an attractive therapeutic strategy due to its long-term colonization ability, which is an essential quality for chronic disease treatment [25]. However, many such bacterial hosts used for therapy lack amenable genetic tools, except for a few model organisms [10]. Thus, since the past decades, many studies have been focusing on developing synthetic genetic parts in various species of bacteria including model organisms of *E. coli*, *L. lactis*, *Lactobacillus* and as well as in intractable microbes such as *Bacteroides* and *Bifidobacterium longum*. In particular, the advancement of high-throughput sequencing techniques allows the construction of a large repertoire of bioparts, such as promoters, ribosome binding sites (RBS) and terminators based on accurate genome-wide information. Such bioparts are the basic constituents required to construct genetic regulation systems [106]. 

#### 3.3.1. Development of Genetic Parts

Genetic parts include native genetic parts that microbes have originally and synthetic genetic parts developed by genetic engineering. Elucidation of native genetic regulation systems is the first step in the development of new genetic parts (Figure 2). Genetic regulation systems comprise several genetic parts: promoters to initiate transcription, RBS to initiate translation and terminators to stop transcription. Promoters are the most frequently used regulatory bioparts, conventionally obtained from the upstream sequence of a gene [107]. For example, bacterial transcription initiation signals and *cis*-regulatory elements are present within hundreds of base pairs upstream of the coding sequence [107]. A large number of promoters were elucidated from the genome-wide transcription start site (TSS) information obtained through differential RNA sequencing (dRNA-seq) [108]. In particular, primary promoter structures, such as −35 and −10 elements, were determined through sequence alignment [109]. In addition, the strength of the endogenous promoters can be modulated by slight modification of the conserved sequence and by changing the spacing between the promoter elements [110]. *Cis*-regulatory motifs can also be elucidated by analyzing the upstream sequence of genes that change their expression in a similar pattern [111]. RBS is a translation initiation signal and its sequence has been widely used for fine-tuning the gene expression level post-transcriptionally [112]. RBS can be identified through ribosome profiling, which footprints the active ribosomes on mRNA [113]. A recent report on a beneficial microbe in the human microbiome, *Eubacterium limosum*, demonstrated RBS identification via ribosome profiling [114]. Based on the RNA-seq data and ribosome profiles, the RBS associated with gas fermentation was identified and the complex post-transcriptional regulation in the gas fermentation pathway was elucidated [114]. 

To develop regulatory bioparts with a broad dynamic range where endogenous regulatory elements cannot reach, synthetic genetic parts can be made from the backbone of endogenous elements. The first step is to chemically synthesize the genetic parts derived from endogenous regulatory elements with few sequence mutations. Simple genetic systems in synthetic biology coupled with fluorescence reporter systems enable rapid characterization of the synthetic bioparts [115,116]. As an example, synthetic promoters and RBSs have been extensively studied in *B. thetaiotaomicron*. Chemically synthesized degenerate promoters and an RBS library based on conserved consensus sequences of *Bacteroides* showed 70-fold higher expression levels than the previously identified strong promoter [116]. As shown in the example, synthetic biology provides tools to develop large-scale genetic parts with various strengths in a relatively short period of time with only a few reported genetic parts. In recent years, genetic parts have also been developed through computational prediction [117]. The computational models are capable of designing a 5- untranslated region (UTR) with the desired transcriptional and translational strength, as demonstrated by the UTR Designer and RBS Calculator [118,119]. As described above, synthetic biology enables rapid development of genetic parts, even in some of the intractable members of the human microbiota. This may facilitate the development of improved therapeutic microbial constructs with a broad dynamic range and high accuracy to sense biomarkers and produce therapeutic molecules.

#### 3.3.2. Genetic Circuits

Genetic circuits are an assembly of genetic parts to convey designed functions (Figure 2) [105]. Thus, well-characterized bioparts are critical for constructing robust genetic circuits. An inducible expression system is a conventional and simplest form of a genetic circuit. The system utilizes a specific transcription factor whose DNA binding strength depends on its target ligand [120]. The *E. coli* lac system is a representative inducible system that has been used for the construction of numerous biological systems from basic to applied sciences for decades [120]. For instance, this simple genetic circuit was applied to eradicate *P. aeruginosa* in response to QS molecules through production of toxins that are under control of the *P. aeruginosa* quorum-specific promoter [121]. In addition, another genetic circuit system is a toggle switch that can maintain either ON-state or OFF-state of the designed function [122]. For example, it was applied to detect tetrathionate, which is a biomarker of gut inflammation. During gut inflammation, tetrathionate activates the β-galactosidase toggle switch from OFF-state to ON-state, so that history of inflammation can be tracked in the later diagnosis [35]. More recently, a CRISPRi-based inducible genetic circuit to detect *V. cholerae* became available [123]. To elaborate, this circuit responds to *V. cholerae*-specific CAI-1; at a low *V. cholera* cell density, CRISPRi inhibits the expression of the reporter system, while at high cell density, single-guide RNA expression is inhibited and the infection is visualized through the reporter system [123]. As shown here, genetic circuits, even in their simplest form, act as robust tools to control and monitor infection. 

To control the expression of multiple genes from multiple stimuli, the design of complex genetic circuits is required [124]. To this end, biological logic gates have been constructed [124]. Various logic gates from the simple AND and OR gates to complex NOR gates have been constructed by combining many bioparts. Among them, the NOR gate has equivalent functional completeness to NAND logic in the silicon industry [125]. Due to the completeness, all logic gates can be implemented with an appropriate combination of NOR gates [125], which was demonstrated by a digital display in *E. coli* [126]. This genetic circuit is incredibly complex in that it contains 63 regulators and spans approximately 76,000 bp. Because it is difficult to design such a complex circuit manually, a computational design and prediction software, named Cello, was first developed [127]. For example, a genetic circuit responsive to bile acid or anhydrotetracycline was constructed in *B. thetaiotaomicron* using Cello [128]. Many therapeutic circuits are introduced into microbes and therapeutic chassis are under examination [53]. However, there are several limitations to overcome. First, synthetic constructs may exert a metabolic burden on their host microbes. Therefore, there can be deleterious compensation processes, such as unexpected mutations and loss of the function, in response to the burden [24]. To solve these problems, a feedback loop regulating the expression intensity of the synthetic construct in response to the metabolic status of the cell was developed [129]. Next, the stability and robustness of synthetic circuits should be thoroughly examined in the dynamic environment of the human body. The last issue is the biocontainment strategy and biosafety. It is particularly important to control and regulate the therapeutic functions of engineered bacteria accurately and prevent unexpected events such as gene transfer and homologous recombination. This can be done with the development of kill switch-like genetic programs to attenuate or kill the engineered bacteria if needed [24].

## 4. Conclusions and Perspectives

The community of microbes that inhabit human body have developed an intimate symbiotic bond with their host. With accumulating evidence suggesting the beneficial roles of microbiome on human health, they are increasingly highlighted as attractive targets for various therapeutic applications [4]. Considering the therapeutic effects of the microbiome itself, probiotic therapy and FMT are implemented for disease treatment [6,130]. Many reports highlight the efficacy of such therapies in providing symptomatic relief in otherwise incurable diseases [18,19]. However, therapeutic effects are not always reliable owing to the lack of extensive understanding regarding the interactions between microbes and the host. For this reason, these strategies often lead to marginal effects or even negative side effects [20]. As an alternative, the therapeutic robustness of synthetic microbes is being examined [24]. 

The use of synthetic biology techniques for the development of biosensors and genetic circuits for the diagnosis and delivery of therapeutic molecules is implemented in members of the human microbiota [24]. To construct a biosensor, known bacterial sensing systems and synthetic sensors are rewired and reconstructed using genetic engineering, allowing noninvasive and real-time monitoring of diseases [33,37]. For disease treatment, the microbial delivery of various therapeutic molecules has been examined. Host proteins, bacterial therapeutic proteins, antigens and synthetic metabolic pathways have been introduced into the host through heterologous production in microbes. Finally, monitoring and therapy could be integrated using genetic circuits composed of a large number of genetic parts [24]. Thus, microbiome-based therapy using synthetic biology is an attractive and promising treatment that can be applied to a variety of diseases, including chronic diseases. Finally, with the advancement in synthetic biology, we expect an increase in the clinical use of engineered microorganisms in the near future. However, there are several shortcomings in such clinical uses of microbes. First, the dynamic range of the synthetic construct should be optimized to match the physiologically relevant levels for successful diagnosis [131]. In addition, stability and robustness should be ensured in the dynamic environment of the human body. Moreover, synthetic constructs can be burdensome for the microbes, resulting in unexpected deleterious compensation processes [24]. Furthermore, for the clinical testing and use of engineered microbes, effective biocontainment systems are essential to prevent gene transfer into and out of the engineered microbes [132]. 

Additionally, for the clinical use of microbiome-based therapy, exact measurements of the dynamic ranges of sensors and therapeutic efficacy are required. Synthetic biology provides a host-mimicking environment platform as an alternative experimental approach to the conventional time-consuming and expensive clinical testing platforms (Figure 2) [133]. Organoids are the most advanced culture platforms that provide physiological and physical environments that are almost similar to those of the host, such as the skin, liver and intestine [134]. Since it is derived from individual human stem cells, it allows the production of personalized organoids system. In addition, genetic manipulation techniques are in place to manipulate the stem cells or organoid systems with relative ease. Although it is regarded as the culture system with the highest degree of resemblance to in vivo environment, it lacks many cellular components such as blood vessels, immune cells and stroma [135]. Furthermore, it has a low reproducibility due to the lack of standardized protocol and technical difficulties [135]. Microfluidic-based devices are another promising test platform [136]. ‘Gut-on-a-chip’ is a representative microfluidic device that provides a human enteric-specific environment [137]. Inside the chip, there is a mucus layer of human intestinal epithelial cells. Unlike organoids, this chip has shown high reproducibility in every testing experiment [135]. As mentioned above, synthetic biology provides promising in vitro testing platforms, which will help improve our understanding of the interaction between the host and microorganisms and enable an easier and faster evaluation of the therapeutic efficacy and safety of devices. 

## Figures and Tables

**Table 1:** Therapeutic functions of engineered microbes.

Functions	Engineered Microbes	Biomarkers	Therapeutic Molecule	Disease Targeted	Host	Ref.
Disease sensors	*E. coli* Nissle	Heme	-	Gastrointestinal bleeding	Swine	[31]
	*E. coli* Nissle	Nitrate	-	Gut inflammation	Mice	[32]
	*E. coli* Nissle	Thiosulfate	-	Colitis	Mice	[33]
	*E. coli* Nissle	Tumors	-	Liver metastasis	Mice	[34]
	*E. coli* NGF-1	Tetrathionate	-	Gut inflammation	Mice	[35]
	*E. coli*	NO, glucose	-	Inflammation, diabetes	Human clinical samples	[36]
	*E. coli*	NO	-	Colitis	Mouse ileum explants	[37]
	*Lactococcus lactis*	CAI-1	-	*Vibrio cholerae* infection	Mice	[38]
	*Lactobacillus reuteri*	AIP-I	-	*Staphylococcus aureus* infection	*In vitro* batch culture	[39]
Heterologous productionsof host proteins	*Bacteroides ovatus*	Xylan	TGF-β1	Colitis	Mice	[40]
	*Bacteroides ovatus*	-	KGF-2	Colitis	Mice	[41]
	*E. coli* Nissle	-	NAPEs	Obesity	Mice	[42]
	*Lactococcus lactis*	-	IL-10	Colitis	Mice	[43]
	*Lactococcus lactis*	-	IL-10	Crohn’s Disease	Patients	[44]
	*Lactococcus lactis*	-	IL-17A	Cancer	Mice	[45]
	*Lactococcus lactis*	-	Heme oxygenase-1	Colitis	Mice	[46]
	*Lactococcus lactis*	-	hTFF1	Oral mucositis	Hamsters	[47]
	*Lactobacillus casei*	-	hLF	Bacterial infection	Mice	[48]
	*Lactobacillus gasseri*	-	GLP-1	Diabetes	Rats	[49]
Heterologous productions of therapeutic proteins	*Bifidobacterium longum*	-	rhMnSOD	Colitis	Mice	[50]
	*E. coli* Nissle	-	CAI-1	*Vibrio cholerae* infection	Mice	[51]
	*E. coli* Nissle	-	Fructose dehydrogenase, mannitol-2-dehydrogenase	Hepatic steatosis	Rats	[52]
	*E. coli* Nissle	AHL	S5 pyocin, E7 lysis protein, DspB	*Pseudomonas aeruginosa* infection	*Caenorhabditis elegans,* mice	[53]
	*E. coli* Nissle	-	HIV-gp41-hemolysin A	HIV	Mice	[54]
	*E. coli*	-	AI-2	Gut microbiota dysbiosis	Mice	[55]
	*E. coli*	CAI-1	YebF-Art-085	*Vibrio cholerae* infection	*In vitro* batch culture	[56]
	*E. coli*	-	Glycosyl-transferase	Diarrhea	Rabbits	[57]
	*E. coli*	-	Invasin, listeriolysin O	Colitis	Mice	[58]
	*E. coli*	-	Myrosinase	Cancer	Cell lines	[59]
	*E. coli*	-	Synthetic adhesins	Cancer	Mice	[60]
	*Lactococcus lactis*	-	Antienterococcal peptides	*Enterococcus faecalis* infection	*In vitro* batch culture	[61]
	*Lactococcus lactis*	-	SCI-59	Diabetes	*In vitro* assay	[62]
	*Lactococcus lactis*	-	Flagellin	Enteropathogen infection	*In vitro* batch culture	[63]
	*Lactococcus lactis, Lactobacillus casei*	-	Elafin	IBD	Cell lines, mice	[64]
	*Lactobacillus jensenii*	-	CV-N	HIV	Simians	[65]
	*Lactobacillus paracasei*	-	Linoleic acid isomerase	Obesity	Mice	[66]
	*Lactobacillus paracasei*	-	Listeria adhesion protein	*Listeria monocytogenes* infection	Cell lines	[67]
	*Salmonella typhimurium*	-	Anhydrotetracycline	Cp53 peptide	Cell lines	[68]
	*Salmonella typhimurium*	-	Flagellin B	Metastatic cancer	Mice	[69]
Synthetic metabolism	*E. coli* Nissle	-	Phe-degradation pathways	Phenylketonuria	Mice, monkeys	[70]
Antigen, antibody induction	*Caulobacter crescentus*	-	Surface-layer protein G	HIV	*In vitro* batch culture	[71]
	*Lactococcus lactis*	-	Anti-TNF nanobody	Colitis	Mice	[72]
	*Lactococcus lactis*	-	Hemagglutinin	Influenza virus infection	Mice	[73]
	*Lactococcus lactis*	-	Ovalbumin	Autoimmune diseases	Mice	[74]
	*Lactococcus lactis*	-	DQ8 gliadin epitope	Celiac disease	Mice	[75]
	*Lactococcus lactis*	-	GAD65, IL-10	Type 1 diabetes	Mice	[76]
	*Lactococcus lactis*	-	LcrV antigen	*Yersinia pseudotuberculosis* infection	Mice	[77]
	*Lactobacillus jensenii*	-	Cyanovirin-N	HIV	Simians	[65]
	*Lactobacillus jensenii*	-	RANTES, C1C5 RNATES	HIV	*In vitro* assay	[78]

Abbreviations: AI-2, autoinducer-2; AIP-I, autoinducer peptide I; AHL, N-acyl homoserine lactone; CAI-1, cholera autoinduer-1; CV-N, cyanovirin-N; DspB, dispersin B; GAD65, glutamic acid decarboxylase; GLP-1, glucagon-like peptide-1; hLF, human lactoferrin; hTFF1, human trefoil factor 1; IBD, inflammatory bowel disease; IL-10, interleukin 10; KGF-2, keratinocyte growth factor-2; LcrV, low-calcium response V; NAPEs, N-acylphosphatidylethanolamines; rhMnSOD, recombinant human manganese superoxide dismutase; SCI-59, single-chain insulin analog; TGF-β1, transforming growth factor-β; TNF, tumor necrosis factor-α. NO, nitric oxide.
